# Online Public Rumor Engagement Model and Intervention Strategy in Major Public Health Emergencies: From the Perspective of Social Psychological Stress

**DOI:** 10.3390/ijerph19041988

**Published:** 2022-02-10

**Authors:** Jiaqi Liu, Jiayin Qi

**Affiliations:** 1 Institute of Journalism and Communication, Chinese Academy of Social Sciences, Beijing 100021, China; liujq@cass.org.cn; 2 Institute of Artificial Intelligence and Change Management, Shanghai University of International Business and Economics, Shanghai 201620, China; 3 Key Laboratory of Trustworthy Distributed Computing and Service (BUPT), Ministry of Education, Beijing University of Posts and Telecommunications, Beijing 100876, China

**Keywords:** public health emergencies, social psychological stress, rumor engagement, rumor intervention, simulation modeling

## Abstract

During major public health emergencies, a series of coupling problems of rumors getting out of control and public psychological imbalance always emerge in social media, which bring great interference for crisis disposal. From the perspective of social psychological stress, it is important to depict the interactive infection law among distinct types of rumor engagers (i.e., advocates, supporters, and amplifiers) under different social psychological stress states, and explore the effectiveness of rumor intervention strategies (i.e., hindering and persuasion) from multiple dimensions, to scientifically predict the situation of public opinion field and guide the public to restore psychological stability. Therefore, this paper constructs an interactive infection model of multiple rumor engagers under different intervention situations based on a unique user-aggregated dataset collected from a Chinese leading online microblogging platform (“Sina Weibo”) during the COVID-19 in 2020. The simulation result shows that (1) in the period of social psychological alarm reaction, the strong level of hindering intervention on the rumor engagers leads to more serious negative consequences; (2) in the period of social psychological resistance, the persuasion and hindering strategies can both produce good outcomes, which can effectively reduce the overall scale of rumor supporters and amplifiers and shorten their survival time in social media; (3) in the period of social psychological exhaustion, rumor intervention strategies are not able to have a significant impact; (4) the greater the intensity of intervention, the more obvious the outcome. Experimental findings provide a solid research basis for enhancing social psychological stress outcomes and offer decision-making references to formulate the rumor combating scheme.

## 1. Introduction

With leading the development of the we-media era, social media recipients are not only news receivers, but also turning into news producers and disseminators. Among we-medium, microblogging is particularly eye-catching. Because it creates an “open” society and lets everyone become their own news outlets. However, the sad thing is that some people take advantage of this and create fake news or spread rumors. It is important to note that rumors, as a kind of unverified news, are even worse than fake news. When an unconfirmed incident is updated on microblogging, many users are not alert and use it as a source of precious information and turn it into “their own”. They “stir-fry” it and “add seasoning” to write posts based on it, reporting as if they were witnesses present at the scene. Therefore, rumors always can be more widened and firmly faked on social media, spread just like accurate news, and confuse public opinion.

Especially during this challenging time of high public health risk, the COVID-19 pandemic has resulted in the extraordinary amount of unverified news that the public often has to manage and the pressures they face against the constant nature of breaking news. The public cannot avoid experiencing a series of issues that threaten their ability to distinguish between truth and fake news and survive the crisis. For example, seeing sudden major infectious diseases and death, the public inevitably lacks a priori knowledge and falls into fear and anxious psychological state [1,2]. The long-term lack of media literacy education also makes it easier for people to engage in rumors in the psychological crises of extremely negative and limited rationality. Thus, along with the persistent battle against the COVID-19 epidemic, the battle against the waves of rumors is equally challenging [3]. In many cases, rumors are even faster and have more serious consequences than the raging coronavirus.

On this front, many scholars have reached a consensus that the preconditions that can prevent rumors are mostly media literacy, the education of critical thinking, and preventing this kind of creation with knowledge [3]. Following this theoretical stream, Hadzialic further explicitly put forward an influential formula of resolving psychological stress and reducing the spread of rumors with media literacy as the core. That is, media literacy (classes “y” divided with outcomes “b”) x rumor(g)–fake news(z) creates = healthy psychological environment (w). There is no doubt that the prior educational process within media literacy is essential. It helps to fundamentally curb the abuse of social media and reduce rumors. Ex-ante media literacy education in many countries is, however, obviously insufficient [4]. Under such a condition, ex-post rumor intervention, as a kind of control and remedial measure which minimizes the losses, also becomes quite critical [3]. However, there is a problem, rumor refutation probably enhances the audience’s trust and support for rumor, that is, the backfire effect [5]. In practice, the backfire effect exacerbates people’s concern that rumor refutation may be counterproductive and leads the social network management department to prefer the hindering strategy (for example, using algorithms to reduce the exposure of incorrect information or shield rumors) to the persuasion strategy (for example, refuting rumors with explaining why) in the process of rumor governance. However, only hindering rumors may lead rumor engagers to believe rumors more because they are not exposed to the truth. In this dilemma, scientifically explaining the mechanism of online rumor engagement and formulating the precise rumor intervention scheme with the consideration of psychological factors are of great significance.

To be more specific, rumor engagement refers to the psychological state of immersion and existence when people interact with unverified news or interpersonal communication around hot events of public opinion in the network situation, which can be externalized and presented in some actual behaviors (such as posting, commenting, sharing, liking, tagging, etc.) after emotional and cognitive processing [6,7]. According to the research conducted by Vaast et al., each engager is naturally given various roles advocate, supporter, and amplifier for the sake of their distinct behavioral characteristics and patterns of social media feature used under the emergency crisis [8]. Inspired, we infer the three kinds of roles infect each other, interact, and form a team in the process of rumors diffusion, which generate a new source of the social force to promote the development of epidemic-related public opinion events. We further summarize the specific differences of rumor engagers, as shown in Table 1.

And, if rumors that get out of control are the external representation of imbalanced social psychology and the tips of the iceberg exposed on the sea surface, most of the contents below the sea surface reflect undisclosed and latent complex social psychology [9]. Especially after being suddenly stimulated by the abnormal development of the epidemic, the public usually has a social and psychological stress response [10,11], and falls into the imbalance and shock of negative psychological emotions such as panic, anxiety, and anger, making the public opinion environment tend to deteriorate. This is the natural function of organisms to constantly adapt to the interference of stressors on the homeostasis of the body. The social psychological stress response generally includes three different stages: social psychological alarm reaction period, social psychological resistance period, and social psychological exhaustion period. In particular, (1) during the social psychological alarm reaction period, the public produces psychological and physiological alarm responses due to the stimulation of stressors, which is easy to form a huge social psychological shock and quickly form rumors. At this time, the flow frequency of unconfirmed information is the highest; (2) during the social psychological resistance period, the public has been adapted under the condition of the continuous effect of stress stimulation, strengthened their resistance to specific stress or stimuli, gradually calmed down, and transitioned from emotional catharsis to rational thought; (3) during the social psychological exhaustion period, to meet the needs of the internal and external environment, the public’s adaptability to stimuli has been formed, but it can no longer be maintained. There are two possible results, exhaustion or recovery. At this time, the public no longer only expresses their opinions around the event itself, and the dissemination of unconfirmed information is restrained [12,13].

During major public health emergencies, in the environment where the epidemic-related rumors collide, the deviant connection actions entail new risks to social psychological health. At the same time, the change of psychological cognition and attitude caused by the transition of social psychological stress response state will, in turn, affect the externalized expression of public opinion and the change of rumor engagement state. In this process, the important role of the crisis management person is to act as a bridge between the damaged external actual world and the threatened internal public psychological world, as well as promote the rumor engagers to transform into rational immune groups. Therefore, researchers and crisis managers should fully judge and guide the development of the diffusion and evolution of rumors from the perspective of social psychological stress of the public.

Building primarily on the 2SI2R model, we propose a conceptual framework that encompasses three components of the rumor mill (i.e., advocates, supporters, and amplifiers), which can infect each other. The framework also depicts three key social psychological stress response states, and we exploit the change of the density of various types of rumor engagers during different periods to assess the effectiveness of diverse rumor intervention strategies (i.e., hindering and persuasion). We examine the relationships between rumor engagement and intervention by using a unique user-aggregated dataset collected from a Chinese leading online social media platform, namely Sina Weibo, through empirical data-driven simulation analyses.

The remainder of this paper is organized as follows: In Section 2, we review some related literature regarding rumor engagement and intervention, and the epidemic model under major public health emergencies. Then, in Section 3, we propose a theoretical model based on the 2SI2R epidemic model that can capture the association between rumor engagement and intervention strategies. In Section 4, we present the empirical case, dataset, and simulation model initialization setting. Section 4 also details a numerical simulation study that is conducted by establishing a theoretical model to evaluate our proposed framework. Section 5 contains the conclusions drawn from the empirical data-driven simulation findings. Last, in Section 6, we discuss the theoretical and managerial implications, and future research directions.

## 2. Related Work

In contrast to the existing studies, we refine the relevant research in the two subsequent sections: Section 2.1 public rumor engagement and intervention and Section 2.2 epidemic model under major public health emergencies.

### 2.1. Public Rumor Engagement and Intervention

Concerning the diffusion trend of network rumors, most of the work is still based on the traditional concept of information security, ignoring the complex psychological factors behind the gossip. Many scholars tend to consider the dynamic evolution process of public rumor engagement as three (i.e., induction, diffusion, and regression) [14] or four (latent, outbreak, stability, and dissipation) objective stages [15]. Few studies have observed the role differences of rumor engagers and systematically captured the infectious law that individuals are assimilated from the ignorant to the engagers (transforming among different types of engagers) and the engagers return to the immune.

In terms of rumor engagement, a large number of researchers have tried to explain why the public is spontaneously engaged in rumor in the public opinion field and why it is engaged in different intensities from the aspects of information dissemination [16,17,18,19,20,21,22], emotional rendering [23,24,25], driving motivation [26,27,28,29,30,31], and have made some progress. However, the investigation on the coupling mechanism between the transition of public psychosocial stress state and rumor engagement is slightly insufficient [9].

Moreover, an emerging body of research on rumor guidance has highlighted the importance of psychological stress intervention. Guiding principles for emergency psychological crisis intervention of COVID-19 infection issued by the National Health Commission of the PRC strongly proposed to “real-time identify and grasp the dynamic changes in the mental health of all groups affected by the epidemic, identify high-risk groups in time, and avoid extreme events and group psychological crisis events”. Experts and scholars also gradually began to summarize the key and core of psychological stress intervention strategies from the emotional, cognitive, and behavioral levels [32,33,34], to reduce the possibility of psychological stress disorder. However, at this stage, the work usually focuses on the health issues of individual psychology and physiology, and the research on the close connection between rumor intervention strategy and social psychological stress has not been in-depth [35].

### 2.2. Epidemic Model under Major Public Health Emergencies

The establishment of a rumor engagement model in the social media environment around major public health emergencies and in-depth analysis of the dynamic mechanism of mutual transformation of various engagement roles under different social psychological stress states are the basis for designing effective rumor intervention strategies.

Many empirical studies show that the dissemination process of rumors is similar to the outbreak process of infectious diseases [36]. In the transmission of infectious disease domain, the epidemic model is one of the most influential conceptual paradigms for understanding this type of interpersonal interaction and infection behavior. Some classical epidemic models (such as SIR) have a rich theoretical basis and strong mathematical rigor. Therefore, modeling the dissemination process of public opinion based on the epidemic model has become a hot research direction in recent years, it has been applied to many major public health emergencies, such as COVID-19, influenza, HIV, SARS, H1N1, and Ebola.

With the in-depth study of disease transmission, scholars pay more attention to two or more infectious diseases. Similarly, considering that two or more kinds of incorrect information may spread in the interpersonal network at the same time, some scholars focus on the interaction and competition mechanism of multiple information. Liu et al. [37] paid attention to the existence of the hesitant group and proposed the SHIR dual competitive rumor dissemination model based on the SIR model. According to the difference of information release time, Zan [14] constructed a DSIR model based on two kinds of rumor content in complex networks. It is noteworthy that Wang, Zhao, and Huang [15] established an information dissemination model 2SI2R containing two kinds of rumors in homogeneous networks. Zhang and Zhu [38] placed 2SI2R in a complex network and analyzed its stability.

2SI2R model provides generalizable insights into the change law of engagement state of people with different levels of influence in the field of public opinion. Previous studies tried to add the external intervention effect on different information based on the epidemic model, which also inspired us to explore the best opportunity, direction, object, and intensity of social psychological guidance to play a role in rumor intervention [39].

Therefore, based on existing research and the improved 2SI2R epidemic model, taking the real events in COVID-19 as an example, we attempt to embed the social psychological stress state in the development of public health emergencies, and construct the interactive infection model of multiple rumor engagers under distinct intervention modes, to deeply reveal the dynamic law of the evolution of rumor engagement behavior, the migration of social and psychological stress, and the effectiveness of intervention strategies. Based on the social psychological stress thought, the research conclusions can provide decision-making theories, strategic methods, and decision support tools for relevant departments and social media platforms to quickly block the diffusion of network rumors and restore the public psychological order.

## 3. Theoretical Model

### 3.1. Analysis of Interactive Infection Process of Multiple Public Rumor Engagers

**Figure 1:**
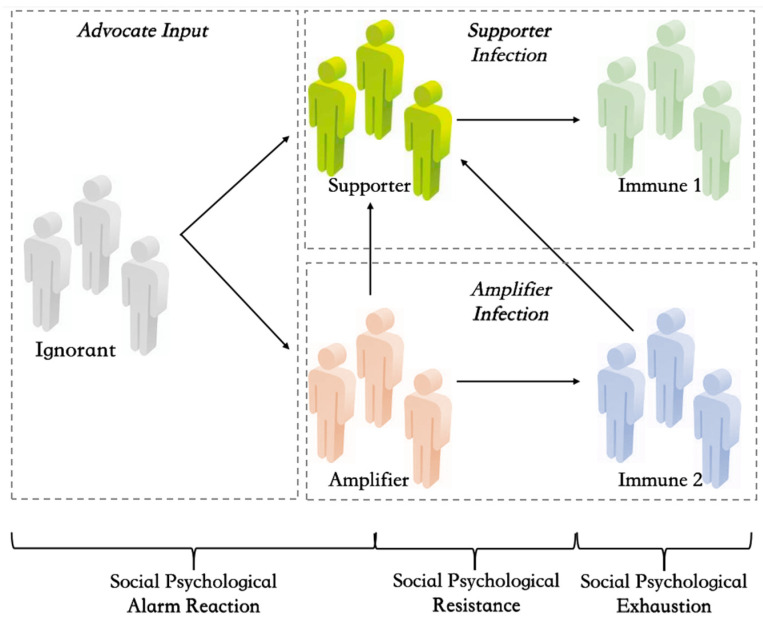
The state transition diagram of the I2S2R interactive infection process of multiple public rumor engagers.

In this section, we discuss the interactive infection process of multiple rumor engagers. Adding to research on the relationships between technology and users as well as the interdependence type among users [8], we attempt to bring deeper insights from a social psychological stress perspective and pay close attention to how the emerging groups of rumor engagers under distinct states of social psychological stress are intricately and mutually related to each other. Our diagram is further developed on the basis of the 2SI2R epidemic model, as depicted in Figure 1.

We firstly consider the whole population in the field of public opinion during major public health crises as a network. In the meantime, we assume that, after the advocates inputting event-related rumors, there are two kinds of rumor engagers in the network, which are called supporter and amplifier. During the advocates spreading rumors in the whole population, the individuals often exist the following potential state: ignorant, who has never heard of event-related rumors and in the state of being easy to be informed of rumors; supporter, who has accepted and spread event-related rumors posted by advocates, and engaged in event-related unconfirmed content generation with stronger influence; amplifier, who has accepted and spread event-related rumors posted by advocates, and not engaged in event-related unconfirmed content generation with weaker influence; immune 1, who has heard event-related rumors but lost interest in engaging as a supporter, and no longer engaged in event-related unconfirmed content generation and rumors dissemination over a while; immune 2, who has heard event-related rumors but lost interest in engaging as an amplifier, and no longer engaged in event-related rumors dissemination over a while. It is worth noting that in the traditional SIR model, it is assumed that the immunized group has permanently immunized antibodies, so it will not be reinfected when in contact with the rumor engagers. However, the 2SI2R model makes an improved assumption that is closer to the actual situation of modern communication. It puts forward that transmitting some hot information may be considered a popular fashion. Therefore, when contacting the rumor engagers with stronger influence, the cured group may be motivated to spread event-related unconfirmed content again and will not be immune permanently [15].

The public rumor engagement role transformation rules are defined as follows.

First, in the period of social psychological alarm reaction, for the sake of the uncertainty, particularity, urgency, and complexity of health crisis events, the ignorants may not have enough knowledge and experience reserves, and the basic resources of rational cognitive assessment are generally weak. As an information source, advocates constantly create and input stimulating and biased event-related information, which makes the ignorants involuntarily fall into a strong inner conflict and tend to blindly connect with advocates. Therefore, when advocates contact ignorants, ignorants may become supporters or amplifiers with a certain probability according to different degrees of instinctive imitation. At this stage, the amount of microblogging information increases explosively in an instant. There are two role transition paths of ignorants in the period of social psychological alarm reaction: from ignorant state to supporter state; from ignorant state to amplifier state.

In the period of social psychological resistance, the public gradually recovers reason and calm after venting negative feelings in the early stage. Some supporters and amplifiers begin to carefully examine the event-related content and lose interest in impulsively creating and diffusing crisis rumors, trying to find the root causes of health crisis and ways to get rid of the crisis through comprehensive thinking. At this time, when supporters contact immune 1, supporters turn into immune 1 with some probability. The relationship between amplifiers and immune 2 is similar. Certainly, some amplifiers with a lower level of rumor engagement may also continue to accumulate negative emotions such as anger, sadness, fear, and anxiety, which have not been fully released before. We assume that when these amplifiers constantly contact supporters, they are likely to turn into supporters. We can understand that supporters may be more influential, more attractive, more infectious than amplifiers, i.e., the supporter is an upgrade version of amplifier in this respect. Therefore, there are three role transition paths at this stage: from supporter state to immune 1 state; from amplifier state to immune 2 state; from amplifier state to supporter state.

Finally, in the period of social psychological exhaustion, with the deep understanding of the health crisis event itself and the continuous emergence of other new hot events, most of the public gradually become calm and stable. The amount of event-related rumors diffusion and generation declined rapidly. However, as set by the 2SI2R infection mechanism, the cured deviant engagers may not be permanently immune. Although some amplifiers engaged in a shallow degree lost their interest in simple rumor diffusion, they did not produce corresponding “antibodies” to deeper event-related content creation and generation. We assume that when these amplifiers contact supporters who are more influential, they may also be infected again to join the team of rumors production and change from their state to supporters with some probability. Therefore, there is one role transition path at this stage: from immune 2 state to secondary supporter state.

### 3.2. Construction of Interactive Infection Model of Multiple Public Rumor Engagers under Intervention

Based on the above analysis of the interactive infection process of multiple public rumor engagers and the summary of role transition paths, we further propose a theoretical model to explore when the intervention strategies (i.e., in the period of social psychological alarm reaction, social psychological resistance, or social psychological exhaustion), what kind of engagers (i.e., supporters or amplifiers) and what direction and intensity of intervention should be taken, which is conducive to effectively control the overall evolution of the rumor engagement in the field of public opinion.

According to the autonomous 2SI2R epidemic model, the engagement process of the public among different roles takes place in an open social media system, and the engagers move in and out remain stable. Because the duration of public health crisis events in real life is generally not long and social media service enterprises probably do not organize large-scale publicity and extreme marketing activities that seriously hinder users from using social networks to obtain information in this circle. Thus, when c represents the coming rate of ignorant for various reasons, and g represents the leaving rate of the population from the public opinion field for being regulated or other natural reasons (such as illness, death, etc.), and c,g∈[0, 1]. We assume that the coming and leaving rates are equal, so c=g. Besides, the public is divided into five groups (i.e., ignorant, supporter, amplifier, immune 1, and immune 2) with densities at time *t* being $I\left(t\right),\;E_{S}\left(t\right),\;E_{A}\left(t\right),\;R_{S}\left(t\right),R_{A}\left(t\right)$, respectively, abbreviated as $I,\;E_{S},E_{A},R_{S},\;R_{A}$.

**Figure 2:**
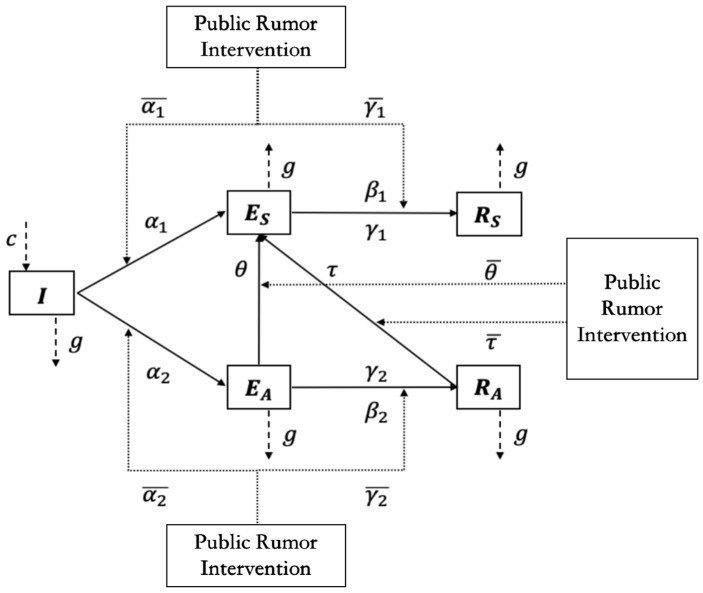
I2S2R interactive infection model of multiple public rumor engagers under different kinds of intervention modes.

Moreover, considering that the social network management department may intervene rumor engagement in three key social psychological stress stages, it can guide and control the evolution of the situation of the rumors. Specifically, the I2S2R interactive infection model of multiple rumor engagers under different kinds of intervention modes is shown in Figure 2, and the meanings of parameters in the model are shown in Table 2.

The average degree of the homogeneous network is denoted by *k*, and $\alpha_{1},\;\alpha_{2},\;\beta_{1},\;\beta_{2},\;\gamma_{1},\;\gamma_{2}$, θ, $\tau \in \left[0,\;1\right],\;\bar{\alpha_{1}},\;\bar{\alpha_{2}},\;\bar{\gamma_{1}},\;\bar{\gamma_{2}},\;\bar{\theta },\;\bar{\tau }\in \left[-1,\;1\right]$. The initial condition is $I+E_{S}+E_{A}+R_{S}+R_{A}=1$. The change rate of the density of ignorant, supporter, amplifier, immune 1, and immune 2 at time *t* are denoted by $\frac{dI}{dt},\;\frac{dE_{S}}{dt},\;\frac{dE_{A}}{dt},\;\frac{dR_{S}}{dt},\;\frac{dR_{A}}{dt}$, respectively. The total number of individuals in the field of public opinion is *N*. According to the dynamic theory, we established the I2S2R interactive infection model of multiple rumor engagers under different kinds of intervention modes based on the above assumptions. The mean-field equations are described as follows [31]:

(1) $$\left\{\begin{array}{c} \frac{dI}{dt}=c-\left(\alpha_{1}-\bar{\alpha_{1}}\right)IE_{S}k-\left(\alpha_{2}-\bar{\alpha_{2}}\right)IE_{A}k-gI\\ \frac{dE_{S}}{dt}=\left(\alpha_{1}-\bar{\alpha_{1}}\right)IE_{S}k-\left(\gamma_{1}-\bar{\gamma_{1}}\right)E_{S}R_{S}k-\beta_{1}E_{S}+\left(\theta -\bar{\theta }\right)E_{A}E_{S}k+\left(\tau -\bar{\tau }\right)R_{A}E_{S}k-gE_{S}\\ \frac{dE_{A}}{dt}=\left(\alpha_{2}-\bar{\alpha_{2}}\right)IE_{A}k-\left(\gamma_{2}-\bar{\gamma_{2}}\right)E_{A}R_{A}k-\beta_{2}E_{A}-\left(\theta -\bar{\theta }\right)E_{A}E_{S}k-gE_{A}\\ \frac{dR_{S}}{dt}=\left(\gamma_{1}-\bar{\gamma_{1}}\right)E_{S}R_{S}k+\beta_{1}E_{S}-gR_{S}\\ \frac{dR_{A}}{dt}=\left(\gamma_{2}-\bar{\gamma_{2}}\right)E_{A}R_{A}k+\beta_{2}E_{A}-\left(\tau -\bar{\tau }\right)R_{A}E_{S}k-gR_{A} \end{array}\right.$$

We suppose $\alpha_{1}{}^{*}=\alpha_{1}-\bar{\alpha_{1}},\;\alpha_{2}{}^{*}=\alpha_{2}-\bar{\alpha_{2}},\;\gamma_{1}{}^{*}=\gamma_{1}-\bar{\gamma_{1}},\;\gamma_{2}{}^{*}=\gamma_{2}-\bar{\gamma_{2}},\;\theta^{*}=\theta -\bar{\theta },\;\tau^{*}=\tau -\bar{\tau }$ and simplify Equations (1) to obtain the following mean-field Equation (2).

(2) $$\left\{\begin{array}{c} \frac{dI}{dt}=c-\alpha_{1}{}^{*}IE_{S}k-\alpha_{2}{}^{*}IE_{A}k-gI\\ \frac{dE_{S}}{dt}=\alpha_{1}{}^{*}IE_{S}k-\gamma_{1}{}^{*}E_{S}R_{S}k-\beta_{1}E_{S}+\theta^{*}E_{A}E_{S}k+\tau^{*}R_{A}E_{S}k-gE_{S}\\ \frac{dE_{A}}{dt}=\alpha_{2}{}^{*}IE_{A}k-\gamma_{2}{}^{*}E_{A}R_{A}k-\beta_{2}E_{A}-\theta^{*}E_{A}E_{S}k-gE_{A}\\ \frac{dR_{S}}{dt}=\gamma_{1}{}^{*}E_{S}R_{S}k+\beta_{1}E_{S}-gR_{S}\\ \frac{dR_{A}}{dt}=\gamma_{2}{}^{*}E_{A}R_{A}k+\beta_{2}E_{A}-\tau^{*}R_{A}E_{S}k-gR_{A} \end{array}\right.$$

Based on the methodology of infectious diseases, the equilibria, local stability, and global stability analysis of the system are reported in Appendix A.

## 4. Empirical Case and Numerical Simulations

Simulation is a quantitative method to create an artificial twin society by constructing a series of mathematical models, to realize the in-depth mining of the truth in the real system and the prediction of the evolution law. This approach offers several advantages such as optimized system design, low experimental costs, low risk of failure, and high predictive power. Relying on the rise of simulation methods and technological progress, the research in the field of crisis management and public decision-making compensates for weaknesses of traditional qualitative methods of the deduction and induction of social laws, to promote intervention strategy design more scientific and humanized. In particular, data-driven based on empirical cases is the source power to make the simulation system close to the real system.

**Figure 3:**
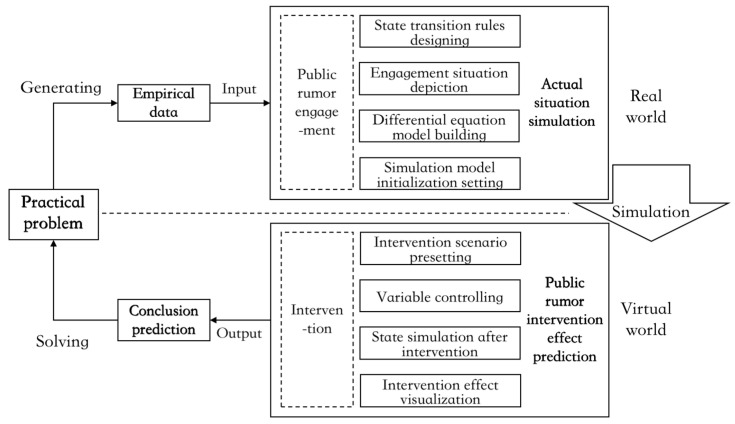
The flow chart of empirical data-driven simulation.

Considering that in complex social networks, the state transition path of rumor engagers cannot be obtained only from the mathematical derivation of differential equations, nor can the effect trend of improving, worsening, or no impact caused by intervention be observed intuitively. In this section, MATLAB is further used for data visualization, data analysis, and numerical calculation to explore the impact of the modes’ change in the direction and intensity of rumor intervention to all kinds of engagers on the trend evolution of the public opinion field under distinct social psychological stress states. The specific simulation process is shown in Figure 3.

### 4.1. Data and Empirical Sample

Health support has been sought by the public from online social media after the outbreak of COVID-19. In addition to the physical symptoms caused by the virus, there are adverse impacts on psychological responses. According to Baidu Index [40], the COVID-19 related issue first appeared in search peak on 25 February 2020, which means that public attention has reached its first climax. This study focuses on the whole life cycle of the first round of primary public opinion hot topics to observe the natural evolution trend of public engagement with little intervention.

The experiment collects a unique dataset for the period by day from a leading zero-threshold Chinese online social media platform, Sina Weibo. The platform features microblogs and is equivalent to Twitter in the US. Users can interact with one another through designed social features, such as following, commenting, posting, sharing, liking, etc., which naturally offers a good opportunity to observe how public opinion takes shape and grows. What is more, Sina Weibo API provides developers with programmatic access to the service. Via the API interface, we can read and write to all aspects of the service including microblogging, comments, users, topics, relations, and much more. To be more specific, searching by keyword surrounding the COVID-19 theme, we crawl the dataset by the API for the period between 22 February 2020 and 6 April 2020 (total 45 days), during which time a total of 194,183 observation users’ engagement data are collected after machine-aided content relevance checking. Each public engagement information includes microblogging ID, text content, the amount of interaction behavior (sharing, commenting, and liking), embedded pictures, videos, publishing time, user ID, etc. According to the research needs, we delete the information that is duplicated, missing more than half of the fields, and irrelevant to the event. T-tests showed that there was no difference between the sample users and the overall population in terms of gender.

Based on the above empirical dataset, considering the significant differences in the performance characteristics of users’ social network service function use and engagement behavior, clustering enables us to group rumor engagers on the basis of similarity in feature use. Specifically, we use density-based spatial clustering of applications with noise (DBSCAN) as a clustering algorithm [8]. In the context of social media, DBSCAN is useful to us because it allows the discovery of nonlinear clusters of uneven sizes. DBSCAN is also robust to noise and outliers. In this study, the configuration of three clusters seems the most appropriate for further analyses. The different clusters, namely advocates, supporters, and amplifiers, emerge many meaningful similarities or differences, as shown in Table 3. The advocate is the smallest cluster (10,403 advocates). It is marked by its high original posting activity (highest average number of original messages) and heavy use of all key features of microblogging (mentions, links, topics). The supporter is a larger cluster (65,600 supporters). It is marked by some original posting activity (moderate average number of original messages) and limited use of all key features of microblogging. At first glance, advocates and supporters may have seemed to differ mostly in their intensity of use. Yet, there are some further distinctions in their patterns of feature use. Compared with advocates, supporters generate many fewer original messages (94.9% less than advocates), mention fewer specific users (36.7% less use of the “@” feature than advocates), engage in the labeling of microblogging through hashtags (52.8% fewer topics) and refer to fewer external sources of information (53.9% fewer links). The amplifier presented a strikingly different pattern of feature use from advocates and supporters. It is also the largest cluster by far (118,180 amplifiers). This cluster is marked by the absence of original posting activity (no original messages) and by its exclusive use of a single feature of microblogging, that of the sharing.

### 4.2. Simulation Model Initialization Setting

For the sake of comparing the simulation results under various rumor intervention modes, it is necessary to reasonably set the proportion of rumor engagers in the benchmark model and the initial values of relevant parameters. We standardize the density of each type of engagers for accurately comparing their transformation trends. Considering that it is difficult to estimate the total number of users who pay attention to target topic in the social network system, and the value of the total number of users do not affect the relative proportion relationship after standardization, referring to the treatment methods of previous research, we assume it as a constant of 100,000 [39]. According to the empirical dataset, at the beginning of the discussion, most users in the social network system are often ignorant, and there are very few supporters and amplifiers. Only a few deviant advocates (such as malicious rumor mongers and network instigators) input event-related rumors. Therefore, we assume parameters $I\left(0\right)=0.9,\;E_{S}\left(0\right)=0.003,\;E_{A}\left(0\right)=0.003,\;R_{S}\left(0\right)=0,\;R_{A}\left(0\right)=0$. Moreover, through estimating the collected empirical data, adjusting according to the model constraints, and referring to the previous literature, we set the parameters in the initial condition as $\alpha_{1}=0.035,\;\alpha_{2}=0.075,\;\beta_{1}=0.03,\;\beta_{2}=0.005,\;\gamma_{1}=0.03,\;\gamma_{2}=0.005,\;\theta =0.005,\;\tau =0.005,\;c=g=0.001,\;k=50$.

**Figure 4:**
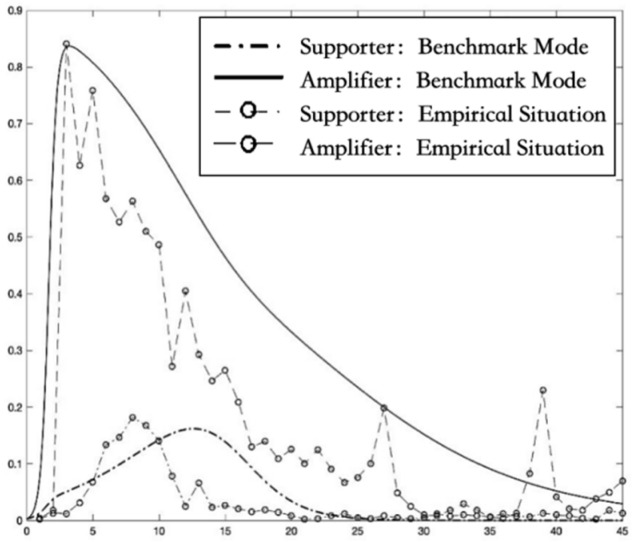
The curves of the density of supporters and amplifiers over time under the empirical situation and benchmark mode.

The function ODE45 and PLOT function in MATLAB software r2018b are used to solve and plot the benchmark model of differential equations (1) without intervention, as shown in Figure 4. The transformation trend of the density of supporters and amplifiers is similar to the real change in the empirical case, meaning that the simulation effect is good.

### 4.3. Numerical Simulation of Rumor Intervention Mode

In this section, six simulation models of rumor intervention are proposed for three different stages of social psychological stress to investigate the influence of factors, that is, the intervention stage, intervention object, intervention direction, and intervention intensity on the survival of the two rumor engager groups. To reflect the intervention effectiveness for each strategy, the variable control method is adopted. Each model only regulates and controls one parameter. There are 24 experimental groups in this simulation experiment. The meaning of the simulation scenario and specific parameter setting of each experimental group are shown in Table 4 and Table 5.

### 4.4. Analysis of the Effect of Rumor Intervention

**Figure 5:**
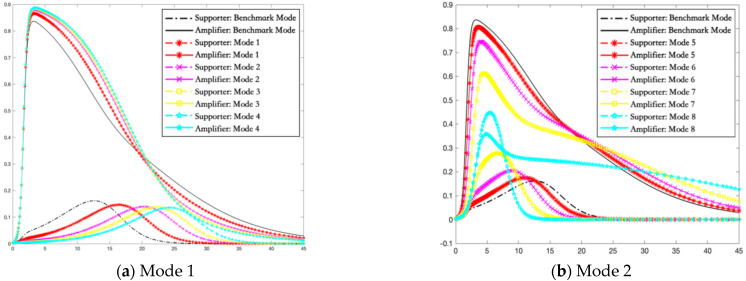
The density of supporters and amplifiers over time in the period of social psychological alarm reaction. (**a**) The variation trends of the proportion of supporters and amplifiers in Mode 1. (**b**) The variation trends of the proportion of supporters and amplifiers in Mode 2.

**Figure 6:**
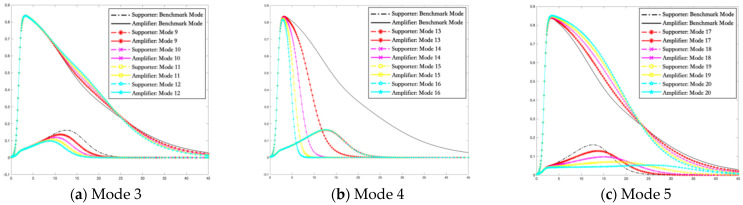
The density of supporters and amplifiers over time in the period of social psychological resistance. (**a**) The variation trends of the proportion of supporters and amplifiers in Mode 3. (**b**) The variation trends of the proportion of supporters and amplifiers in Mode 4. (**c**) The variation trends of the proportion of supporters and amplifiers in Mode 5.

**Figure 7:**
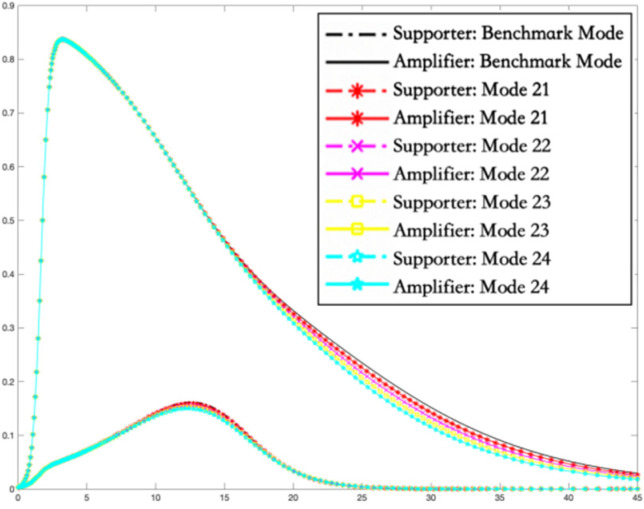
The density of supporters and amplifiers over time in Mode 6 in the period of social psychological exhaustion.

In this section, simulations are carried out using the Runge–Kutta method to solve the differential equations of the I2S2R interactive infection model of multiple rumor engagers. The variation trends of the proportion of supporters and amplifiers in different rumor intervention modes are shown in Figure 5, Figure 6 and Figure 7.

#### 4.4.1. The Influence of the Rumor Intervention Strategy during the Social Psychological Alarm Reaction Period

Figure 5a shows that, in the case of mode 1, compared with the benchmark model, in the period of social psychological alarm reaction, the hindering intervention in the transformation from ignorant to deviant supporter can delay the time when the number of deviant supporters reaches the peak. It reflects the property of rumor intervention that hindering strategy is more intense; the speed of transmission rate of supporters is slower. However, the total number of supporters does not decrease but the peak value of the number of amplifiers increases significantly overall. Precisely, with increasing intensity of hindering intervention, the peak of the rumor amplifiers is higher and the descent rate to fall back to zero is larger. Therefore, if the crisis management department blocks the deviant supporters in the period of social psychological alarm reaction, it may seem to be conducive to reducing the blind and impulsive content creation of the deviant supporters in the early stage. In essence, it does not completely solve the problem and improve the current situation. Moreover, the direct suppression of deviant supporters will stimulate more deviant amplifiers and pose a greater threat to the stability of the social system. In short, the hindering intervention on the deviant supporters leads to more serious negative consequences in the period of social psychological alarm reaction.

Figure 5b describes how the density of supporters and amplifiers changes over time along with the different hindering intervention intensity under Mode 2. In Figure 5b, we can observe that the peak of amplifiers becomes lower with the increase of the peak value of supporters after hindering intervention measures had been taken in the transformation from ignorant to deviant amplifier during the social psychological alarm reaction period. The more intense the hindering intervention, the less the number of amplifiers engaging in the topic discussion. At the same time, the peak of the rumor supporters is higher and the descent rate to fall back to zero is larger, which illustrates if the crisis management department cut off the deviant amplifiers in the early stage, although the intervention measure can weaken the diffusion of deviation information currently, it could lead to more severe negative consequences subsequently, such as expanding the scale of deviant supporters and accelerating their aggregation speed. In the circumstances, large-scale new original and contagious rumors outbreak rapidly in the field of public opinion, aggravating the difficulty of users to remain rational and sober in the period of social psychological alarm reaction. Combined with the results in Figure 5a,b in the period of social psychological alarm reaction, the hindering intervention of deviant supporters and amplifiers may trigger adverse consequences.

#### 4.4.2. The Influence of the Rumor Intervention Strategy during the Social Psychological Resistance Period

Figure 6a depicts how the density of supporters and amplifiers changes over time during the social psychological resistance period along with the different persuasion intervention intensity under Mode 3. In Figure 6a, we can note that supporters die out faster with the increase of persuasion intervention intensity. More and more supporters turn immune due to the mild and flexible nudging. The time to reach the balance is the shortest when the strength of persuasion intervention is the biggest. The increase in the persuasion intervention intensity also results in a lower peak of the rumor supporters and a smaller descent rate to fall back to zero in this paper. What is more, there is no significant change in the survival status of deviant amplifiers. Namely, compared with the hindering strategy in Mode 1, the persuasion intervention in Mode 3 does not resolve the potential crisis of generating deviant supporters at the cost of increasing the total density of amplifiers. Therefore, it provides us with a quick way to make deviant supporters disappear is to increase the persuasion intervention intensity in the period of social psychological resistance, rather than in the period of social psychological alarm reaction.

In Figure 6b, in Mode 4, the increase of the strength of persuasion intervention in the transformation from amplifiers to immune during the social psychological resistance period can weaken the expansion of amplifiers. Exactly, the peak of the rumor amplifiers is not obviously changed but the descent rate to fall back to zero is larger due to the mild and flexible nudging. The strategy also significantly shortens the time for deviant amplifiers to exist in the field of public opinion and makes a large number of amplifiers die out and turn into immune faster. Comparing Mode 2 and Mode 4, it is found that although the blocking of amplifiers in the period of social psychological alarm reaction may reduce their peak value, it quickly increases the proportion of deviant supporters at the same time, resulting in a more serious emergency burden. However, in the period of social psychological resistance, although the persuasion of amplifiers cannot reduce their peak value, it is more conducive to reducing the engagement scale of amplifiers and quickly eliminating the group of irrational amplifiers. Without the help of amplifiers, the rumors created by supporters and advocates further lose great development space correspondingly, thus decreasing the level of the overall destructiveness of the event. Hence, with regard to amplifiers, the persuasion in the social psychological resistance period is far more effective than the blocking in the social psychological alarm reaction period.

As can be seen from Figure 6c, in the case of Mode 5, the hindering intervention in the transformation from amplifiers to supporters can significantly reduce the peak value of deviant supporters. Supporters slowly reach the peak in a longer time under smaller of the transmissions rate. Strictly, the stronger the hindering intervention, the fewer rumor supporters exist at the same time, but the longer they exist. In addition, the implementation of Mode 5 has a little negative impact on the scale of amplifiers. Its peak increased slightly.

Combined with the situation reflected from Figure 6a–c, the intervention decision-making in the period of social psychological resistance is key. Once the strategy is implemented properly, it will not only hinder the gathering of deviant supporters but also facilitate the rapid disbanding of deviant amplifiers groups. Moreover, no matter which strategy is applied in the period of social psychological resistance, the effect is better than that in the alarm reaction period.

#### 4.4.3. The Influence of the Rumor Intervention Strategy during the Social Psychological Exhaustion Period

Figure 7 depicts that, in the case of Mode 6, in the period of social psychological exhaustion, the hindering intervention in the transformation of immune to secondary supporters will not have a significant impact, and the overall trend has no significant change. It illustrates that for the secondary rumor engagers who have temporarily recovered their reason, the intervention effect in the social network platform is no longer significant. For such people, they may need more professional psychotherapy.

## 5. Conclusions

In this paper, we propose a new research framework inspired by psychological stress response and 2SI2R theory to understand the interactive infection law among distinct types of rumor engagers (i.e., advocates, supporters, and amplifiers) under different social psychological stress states. The simulation results show that there is a significant positive correlation between the intensity of rumor intervention and the outcome. Concerning the distinct social psychological stress states, the strong hindering intervention on the deviant engagers (specifically, supporters and amplifiers) leads to more serious negative consequences in the period of social psychological alarm reaction. Conversely, in the period of social psychological resistance, the persuasion and hindering intervention can both produce good outcomes, which can effectively reduce the overall scale of deviant supporters and amplifiers and shorten their existence time in the field of public opinion. In addition, in the period of social psychological exhaustion, rumor intervention is not easy to have a significant impact.

## 6. Discussion and Future Directions

### 6.1. Theoretical Contributions

This study contributes to the existing body of literature in the following ways.

First, in addition to the physical symptoms caused by the virus, there are adverse impacts on psychological responses. Adequate mental health support should be paid more attention by the crisis management department from online social media. Previous studies on rumor intervention usually focused on the analysis of crisis communication content and rhetorical discourse [17,41], governance structure configuration [42], sentiment variations interpreting [43,44], but in fact, communication strategies need to be more carefully designed. According to the stage of social psychological stress, this study divides crisis events into different periods, which overcomes the limitations of most studies taking public involvement heat and development process as the division standard from the external perspective. To a certain extent, it integrates the boundary of rumors and psychological responses research system, which provides the foundation for future researchers.

Second, this work clarifies the characteristics of different engaged roles in the rumor field, as well as their different action preferences and state transition paths. Many recent studies concerning rumor intervention often take hindering measures for a small number of key advocates with potential deviation characteristics from the source. However, vast numbers of supporters and amplifiers instantly activated by advocates in the social media scene have seldom been observed, which are the core constituent subject and evolutionary power of the field of public opinion. Thus, based on the consideration of supporters and amplifies, we further promote the refined research pattern of rumor intervention objects and make intervention strategy more accurate.

Third, this paper expands the scope of the empirical data-driven simulation research paradigm to the rumor research context. Although many scholars have called for making full use of social media channels to exert positive energy, increasing the transparency of the public opinion field in the system, and providing timely and accurate information to the whole society [17]. However, it is still not clear that ”when to speak”, ”to whom”, and ”how much volume” can more effectively guide the trend of rumors now. Using simulation experiments, we further explicitly depict the evolution trend of the rumor engagers and the dynamic outcomes of public infection under the influence of different rumor intervention modes, which uncovered a new path in the rumor intervention domain.

### 6.2. Suggestions for the Healthy Development of Social Media Platforms during Major Public Health Crisis

Yielding several important contributions to actual practice, the empirical findings provide real and applied value for rumor governance department and social media platform managers in creating unique healthy development strategies of public opinion field through understanding the law of psychosocial stress response and enhancing crisis communication efficiency in the context of major public health emergencies.

First, in the period of social psychological alarm reaction, our empirical results suggest that the rumor governance department should not forcefully shield rumors and should avoid escalating the conflict, causing panic among the population.

Second, in the period of social psychological resistance, the crisis management department should arouse the resonance of the public in a gentle way and jointly maintain the overall social stability at the internal psychological level. From a practical standpoint, the online fact-checking function launched by Facebook provides a reference value for rumor intervention strategy development in the manner of persuasion. For the false information that has been retweeted in large quantities, Facebook does not simply delete it but covers a gray module of ”False Information” authentication on the original tweet. Social media users can still choose the elimination module, view the original post, and then click ”see why” to see the official explanation of why it is a rumor.

Third, in the period of social psychological exhaustion, it should strengthen the positive energy transmission to the field of public opinion and correct the inherently biased cognition. Given that there is still the possibility of people getting engaged in rumors again, it would be more proper for social media platform managers to launch more rumor combating and explanation functions timely to avoid people deepening their wrong understanding.

### 6.3. Limitations and Future Work

This study still has some limitations. First, although our research examines the infection law of three particular types of rumor engagers and social network rumor diffusion trend on the microblogging platform, other social media contexts are also common (WeChat, TikTok, etc.). Based on our results, it is considered to further verify the effectiveness of the rumor intervention strategy in other social media situations in the future. Second, given that our study mainly focuses on interaction modes and infection paths of different groups at the macro level, we do not uncover the impact of interpersonal coupling network and trust factors on public engagement in decision making at the microlevel. There is evidence from a study that individual engagement behavior may be influenced by friends or the same kind to a great extent [45,46]. Thus, the further exploration of friend relationships and trust effects on rumor engagement should also be taken into account. Third, on the rumor governance side, this study focuses on developing ex-post short-term remedies after rumors bruiting. However, interventions admittedly come late and maybe only dispel in a half of way rumors comparing what can be done with the ex-ante educational process within media literacy. Therefore, in the future, we plan to expand our research to prior media literacy education. It is essential to explore the optimum combination of long-term media literacy education, knowledge construction, critical thinking training, sophisticated search, processing of information collected in an online social media environment, and short-term rumor intervention, to comprehensively enhance people’s rumor identification ability in major public health emergencies.

## Figures and Tables

**Table 1:** The characteristics of rumor engagers.

	Advocates	Supporters	Amplifiers
Actions in rumor engagement	Initiate collective engagement activities; Stimulate the interest of supporters and amplifiers; Lead content creation and rumors output.	After being awakened by the advocates, follow up and support the activities of the advocates in the rumor engagement action in time; Process the unconfirmed content materials provided by the advocates and engage in content creation and rumors output.	Spread the unconfirmed content created by advocates and supporters but do not create any new rumors; Scale-up and maintain over time the momentum of rumor engagement activities.
Scale	Relatively less	Less	Many
Frequency and intensity of social media use	Heavy users	Moderate users	Moderate users
Patterns of feature use	All key features (such as posting, commenting, sharing, liking, tagging, etc.)	All key features, especially sharing and tagging	Only sharing feature
Infection effect	With strong emotional penetration, high topic sensitivity, and strong attraction of published content, it is easy to attract the general attention of the public opinion field.	Strong emotional penetration, high topic sensitivity, strong attraction of published content, and easy to attract the attention of other users.	Only spread, no creation, weak emotional penetration, weak topic sensitivity, and received the least attention alone.
Reciprocal interdependence among roles	Advocates initiate, guide, and rekindle the rumor engagement; Supporters qualify the public rumor engagement; amplifiers scale the rumor engagement by further circulating others’ unconfirmed content and sustaining the momentum.		

**Table 2:** The meaning of the parameters in the model.

Parameter	Psychosocial Stress Stage	Parameter Meaning
$\alpha_{1}$	Social psychological alarm reaction	The transmission probability from ignorant to supporter
$\alpha_{2}$	Social psychological alarm reaction	The transmission probability from ignorant to amplifier
$\beta_{1}$	Social psychological resistance	The transmission probability from supporter to immune 1 due to contacts
$\beta_{2}$	Social psychological resistance	The transmission probability from amplifier to immune 2 due to contacts
$\gamma_{1}$	Social psychological resistance	The transfer rate from supporter to immune 1 due to forgetting mechanism
$\gamma_{2}$	Social psychological resistance	The transfer rate from amplifier to immune 2 due to forgetting mechanism
θ	Social psychological resistance	The transmission probability from amplifier to supporter
τ	Social psychological exhaustion	The transmission probability from immune 2 to supporter
$\bar{\alpha_{1}}$	Social psychological alarm reaction	During the transmission of ignorant to supporter, a certain interven-tion role $\bar{\alpha_{1}}$ is applied to hinder the generation of supporter
$\bar{\alpha_{2}}$	Social psychological alarm reaction	During the transmission of ignorant to amplifier, a certain interven-tion role $\bar{\alpha_{2}}$ is applied to hinder the generation of amplifier
$\bar{\gamma_{1}}$	Social psychological resistance	During the transmission of supporter to immune 1, a certain inter-vention role $\bar{\gamma_{1}}$ is applied to promote the disappearance of supporter
$\bar{\gamma_{2}}$	Social psychological resistance	During the transmission of amplifier to immune 2, a certain interven-tion role $\bar{\gamma_{2}}$ is applied to promote the disappearance of amplifier
$\bar{\theta }$	Social psychological resistance	During the transmission of amplifier to supporter, a certain intervention role $\bar{\theta }\;$ is applied to hinder the generation of supporter
$\bar{\tau \;}$	Social psychological exhaustion	During the transmission of immune 2 to supporter, a certain inter-vention role $\bar{\tau \;}\;$ is applied to hinder the generation of secondary supporter

**Table 3:** Descriptive statistics of role characteristics of engagers.

Role	Number of People	Number of Original Messages	Number of Original Mentions	Number of Original Topics	Number of Original External Links
Advocate	10,403	34.571	0.305	1.022	1.079
Supporter	65,600	1.733	0.193	0.482	0.497
Amplifier	118,180	0.000	0.000	0.000	0.000

**Table 4:** The meaning of the rumor intervention scenario.

Mode	Scenario	Adjustable Parameter	Intervention Stage	Intervention Object	Intervention Direction	Intervention Intensity *
Mode 1	Scenario 1	$\bar{\alpha_{1}}$	Social psychological alarm reaction	Ignorant—> Supporter	Hindering	Level 1
	Scenario 2	$\bar{\alpha_{1}}$	Social psychological alarm reaction	Ignorant—> Supporter	Hindering	Level 2
	Scenario 3	$\bar{\alpha_{1}}$	Social psychological alarm reaction	Ignorant—> Supporter	Hindering	Level 3
	Scenario 4	$\bar{\alpha_{1}}$	Social psychological alarm reaction	Ignorant—> Supporter	Hindering	Level 4
Mode 2	Scenario 5	$\bar{\alpha_{2}}$	Social psychological alarm reaction	Ignorant—> Amplifier	Hindering	Level 1
	Scenario 6	$\bar{\alpha_{2}}$	Social psychological alarm reaction	Ignorant—> Amplifier	Hindering	Level 2
	Scenario 7	$\bar{\alpha_{2}}$	Social psychological alarm reaction	Ignorant—> Amplifier	Hindering	Level 3
	Scenario 8	$\bar{\alpha_{2}}$	Social psychological alarm reaction	Ignorant—> Amplifier	Hindering	Level 4
Mode 3	Scenario 9	$\bar{\gamma_{1}}$	Social psychological resistance	Supporter—> Immune 1	Persuasion	Level 1
	Scenario 10	$\bar{\gamma_{1}}$	Social psychological resistance	Supporter—> Immune 1	Persuasion	Level 2
	Scenario 11	$\bar{\gamma_{1}}$	Social psychological resistance	Supporter—> Immune 1	Persuasion	Level 3
	Scenario 12	$\bar{\gamma_{1}}$	Social psychological resistance	Supporter—> Immune 1	Persuasion	Level 4
Mode 4	Scenario 13	$\bar{\gamma_{2}}$	Social psychological resistance	Amplifier—> Immune 2	Persuasion	Level 1
	Scenario 14	$\bar{\gamma_{2}}$	Social psychological resistance	Amplifier—> Immune 2	Persuasion	Level 2
	Scenario 15	$\bar{\gamma_{2}}$	Social psychological resistance	Amplifier—> Immune 2	Persuasion	Level 3
	Scenario 16	$\bar{\gamma_{2}}$	Social psychological resistance	Amplifier—> Immune 2	Persuasion	Level 4
Mode 5	Scenario 17	$\bar{\theta }$	Social psychological resistance	Amplifier—> Supporter	Hindering	Level 1
	Scenario 18	$\bar{\theta }$	Social psychological resistance	Amplifier—> Supporter	Hindering	Level 2
	Scenario 19	$\bar{\theta }$	Social psychological resistance	Amplifier—> Supporter	Hindering	Level 3
	Scenario 20	$\bar{\theta }$	Social psychological resistance	Amplifier—> Supporter	Hindering	Level 4
Mode 6	Scenario 21	$\bar{\tau }$	Social psychological exhaustion	Immune 2—> Supporter	Hindering	Level 1
	Scenario 22	$\bar{\tau }$	Social psychological exhaustion	Immune 2—> Supporter	Hindering	Level 2
	Scenario 23	$\bar{\tau }$	Social psychological exhaustion	Immune 2—> Supporter	Hindering	Level 3
	Scenario 24	$\bar{\tau }$	Social psychological exhaustion	Immune 2—> Supporter	Hindering	Level 4

* The intervention intensity gradually increases from Level 1 to Level 4, with Level 1 being the weakest and Level 4 being the strongest.

**Table 5:** Parameter setting of rumor intervention scenario.

Mode	Scenario	$\alpha_{1}$	$\bar{\alpha_{1}}$	$\alpha_{2}$	$\bar{\alpha_{2}}$	$\gamma_{1}$	$\bar{\gamma_{1}}$	$\gamma_{2}$	$\bar{\gamma_{2}}$	θ	$\bar{\theta }$	τ	$\bar{\tau }$
Non	Benchmark	0.035	0	0.075	0	0.03	0	0.005	0	0.005	0	0.005	0
Mode 1	Scenario 1	0.035	0.01	0.075	0	0.03	0	0.005	0	0.005	0	0.005	0
	Scenario 2	0.035	0.02	0.075	0	0.03	0	0.005	0	0.005	0	0.005	0
	Scenario 3	0.035	0.025	0.075	0	0.03	0	0.005	0	0.005	0	0.005	0
	Scenario 4	0.035	0.03	0.075	0	0.03	0	0.005	0	0.005	0	0.005	0
Mode 2	Scenario 5	0.035	0	0.075	0.01	0.03	0	0.005	0	0.005	0	0.005	0
	Scenario 6	0.035	0	0.075	0.02	0.03	0	0.005	0	0.005	0	0.005	0
	Scenario 7	0.035	0	0.075	0.03	0.03	0	0.005	0	0.005	0	0.005	0
	Scenario 8	0.035	0	0.075	0.04	0.03	0	0.005	0	0.005	0	0.005	0
Mode 3	Scenario 9	0.035	0	0.075	0	0.03	−0.01	0.005	0	0.005	0	0.005	0
	Scenario 10	0.035	0	0.075	0	0.03	−0.02	0.005	0	0.005	0	0.005	0
	Scenario 11	0.035	0	0.075	0	0.03	−0.03	0.005	0	0.005	0	0.005	0
	Scenario 12	0.035	0	0.075	0	0.03	−0.04	0.005	0	0.005	0	0.005	0
Mode 4	Scenario 13	0.035	0	0.075	0	0.03	0	0.005	−0.01	0.005	0	0.005	0
	Scenario 14	0.035	0	0.075	0	0.03	0	0.005	−0.02	0.005	0	0.005	0
	Scenario 15	0.035	0	0.075	0	0.03	0	0.005	−0.03	0.005	0	0.005	0
	Scenario 16	0.035	0	0.075	0	0.03	0	0.005	−0.04	0.005	0	0.005	0
Mode 5	Scenario 17	0.035	0	0.075	0	0.03	0	0.005	0	0.005	0.001	0.005	0
	Scenario 18	0.035	0	0.075	0	0.03	0	0.005	0	0.005	0.002	0.005	0
	Scenario 19	0.035	0	0.075	0	0.03	0	0.005	0	0.005	0.003	0.005	0
	Scenario 20	0.035	0	0.075	0	0.03	0	0.005	0	0.005	0.004	0.005	0
Mode 6	Scenario 21	0.035	0	0.075	0	0.03	0	0.005	0	0.005	0	0.005	0.001
	Scenario 22	0.035	0	0.075	0	0.03	0	0.005	0	0.005	0	0.005	0.002
	Scenario 23	0.035	0	0.075	0	0.03	0	0.005	0	0.005	0	0.005	0.003
	Scenario 24	0.035	0	0.075	0	0.03	0	0.005	0	0.005	0	0.005	0.004

## Data Availability

Not applicable.

## Appendix A. Details of Model Analysis: Equilibria and Stability Analysis

Based on the methodology of infectious diseases, the infection-free equilibrium point in the system refers to the solution of the differential equations when the event-related rumors do not spread or the spread end. Non-infection-free equilibrium point ${Ε}_{0}$ refers to the solution of the differential equations in the presence of event-related rumors, which means that the connective engagement action continues, and the public engagement tends to be stable. We also manage to define the basic reproduction number $R_{0}$, indicating the number of people infected by a deviant engager during the average rumor engagement period when all are susceptible at the initial stage of event-related rumors diffusion. Usually, $R_{0}$ = 1 can be used as an important threshold to judge whether event-related rumors have diffusion ability. In this section, we investigate the local stability and global stability of the basic reproduction number $R_{0}$ and infection-free equilibrium point ${Ε}_{0}$ for system (2).

**Theorem** **A1.**
*The system (2) has a unique infection-free equilibrium point ${Ε}_{0}=\left(I_{0},\;E_{S0},\;E_{A0},\;R_{S0},\;R_{A0}\right)=\left(1,\;0,\;0,\;0,\;0\right)$.*

**Proof** **of** **Theorem A1.** To calculate the equilibrium point of the system (2), it can be set as following:

(A1) $$\left\{\begin{array}{c} \frac{dI}{dt}=c-\alpha_{1}{}^{*}IE_{S}k-\alpha_{2}{}^{*}IE_{A}k-gI=0\\ \frac{dE_{S}}{dt}=\alpha_{1}{}^{*}IE_{S}k-\gamma_{1}{}^{*}E_{S}R_{S}k-\beta_{1}E_{S}+\theta^{*}E_{A}E_{S}k+\tau^{*}R_{A}E_{S}k-gE_{S}=0\\ \frac{dE_{A}}{dt}=\alpha_{2}{}^{*}IE_{A}k-\gamma_{2}{}^{*}E_{A}R_{A}k-\beta_{2}E_{A}-\theta^{*}E_{A}E_{S}k-gE_{A}=0\\ \frac{dR_{S}}{dt}=\gamma_{1}{}^{*}E_{S}R_{S}k+\beta_{1}E_{S}-gR_{S}=0\\ \frac{dR_{A}}{dt}=\gamma_{2}{}^{*}E_{A}R_{A}k+\beta_{2}E_{A}-\tau^{*}R_{A}E_{S}k-gR_{A}=0 \end{array}\right.$$

 □

Calculating the Equation (A1), we obtain that ${Ε}_{0}=\left(1,\;0,\;0,\;0,\;0\right)$. Thus, Theorem A1 is proved.

**Theorem** **A2.**
*When $R_{0}\leq 1$, the infection-free equilibrium point ${Ε}_{0}\left(1,\;0,\;0,\;0,\;0\right)$ is locally asymptotically stable and when $R_{0}>1$, the infection-free equilibrium point ${Ε}_{0}\left(1,\;0,\;0,\;0,\;0\right)$ is unstable.*

**Proof** **of** **Theorem A2.** The Jacobian matrix of the system (2) at the infection-free equilibrium ${Ε}_{0}\left(1,\;0,\;0,\;0,\;0\right)$ is

(A2) $$J\left({Ε}_{0}\right)=\left[\begin{array}{ccccc} -g & -\alpha_{1}{}^{*}k & -\alpha_{2}{}^{*}k & 0 & 0\\ 0 & \alpha_{1}{}^{*}k-\beta_{1}-g & 0 & 0 & 0\\ 0 & 0 & \alpha_{2}{}^{*}k-\beta_{2}-g & 0 & 0\\ 0 & \beta_{1} & 0 & -g & 0\\ 0 & 0 & \beta_{2} & 0 & -g \end{array}\right]$$

 □

We illustrate the characteristic equation of matrix $J\left({Ε}_{0}\right)$ as

(A3) $$\begin{array}{l} \left|J\left({Ε}_{0}\right)-\lambda Ε\right|\\ =\left[\begin{array}{ccccc} -g-\lambda & -\alpha_{1}{}^{*}k & -\alpha_{2}{}^{*}k & 0 & 0\\ 0 & \alpha_{1}{}^{*}k-\beta_{1}-g-\lambda & 0 & 0 & 0\\ 0 & 0 & \alpha_{2}{}^{*}k-\beta_{2}-g-\lambda & 0 & 0\\ 0 & \beta_{1} & 0 & -g-\lambda & 0\\ 0 & 0 & \beta_{2} & 0 & -g-\lambda \end{array}\right] \end{array}\;={\left(-g-\lambda \right)}^{3}\left(\alpha_{1}{}^{*}k-\beta_{1}-g-\lambda \right)\left(\alpha_{2}{}^{*}k-\beta_{2}-g-\lambda \right)$$

According to the Routh–Hurwitz judgment and the method from Samsuzzoha, Singh, and Lucy [47], we can obtain the basic reproduction number

(A4) $$R_{0}=max\left\{\frac{\alpha_{1}{}^{*}k}{\beta_{1}+g},\frac{\alpha_{2}{}^{*}k}{\beta_{2}+g}\right\}$$

When $R_{0}$ ≤ 1, the characteristic roots are both negative roots. In summary, we finally acquire that, when $R_{0}$ ≤ 1, the infection-free equilibrium point ${Ε}_{0}$ (1,0,0,0,0) is locally asymptotically stable, and if $R_{0}$ > 1, the infection-free equilibrium point ${Ε}_{0}$ (1,0,0,0,0) is unstable.

**Theorem** **A3.**
*The infection-free equilibrium point* ${Ε}_{0}$ *is global asymptotically stable if* $R_{0}$ *≤ 1.*

**Proof** **of** **Theorem A3.** Considering the Lyapunov function of the following form

(A5) $$L\left(t\right)=aE_{S}+bE_{A}$$

 □

Here, $a=\frac{\gamma_{2}{}^{*}}{(\beta_{1}+g)\left(\beta_{2}+g\right)},\;b=\frac{m\tau^{*}}{(\beta_{1}+g)\left(\beta_{2}+g\right)}$, where M is a large enough integer and satisfi-ed $m\tau^{*}\geq \gamma_{2}{}^{*}$.

We now calculate the time derivative of *L(t)* along with the solutions of Equation (2). It can be seen as follows:

(A6) $$\begin{array}{cc} \frac{dL}{dt} & =\frac{\gamma_{2}{}^{*}}{(\beta_{1}+g)\left(\beta_{2}+g\right)}\left(\alpha_{1}{}^{*}IE_{S}k-\gamma_{1}{}^{*}E_{S}R_{S}k-\beta_{1}E_{S}+\theta^{*}E_{A}E_{S}k+\tau^{*}R_{A}E_{S}k-gE_{S}\right)\\ & +\frac{m\tau^{*}}{(\beta_{1}+g)\left(\beta_{2}+g\right)}\left(\alpha_{2}{}^{*}IE_{A}k-\gamma_{2}{}^{*}E_{A}R_{A}k-\beta_{2}E_{A}-\theta^{*}E_{A}E_{S}k-gE_{A}\right)\\ & =\left[\frac{\gamma_{2}{}^{*}}{\beta_{2}+g}\left(\frac{\alpha_{1}{}^{*}IE_{S}k}{\beta_{1}+g}-\frac{\beta_{1}E_{S}+gE_{S}}{\beta_{1}+g}\right)+\frac{\gamma_{2}{}^{*}\tau^{*}R_{A}E_{S}k}{(\beta_{1}+g)\left(\beta_{2}+g\right)}-\frac{\gamma_{2}{}^{*}\gamma_{1}{}^{*}E_{S}R_{S}k}{(\beta_{1}+g)\left(\beta_{2}+g\right)}\right]\\ & +\left[\frac{m\tau^{*}}{\beta_{1}+g}\left(\frac{\alpha_{2}{}^{*}IE_{A}k}{\beta_{2}+g}-\frac{\beta_{2}E_{A}+gE_{A}}{\beta_{2}+g}\right)-\frac{m\tau^{*}\gamma_{2}{}^{*}E_{A}R_{A}k}{(\beta_{1}+g)\left(\beta_{2}+g\right)}\right]\\ & +\left[\frac{\gamma_{2}{}^{*}-m\tau^{*}}{(\beta_{1}+g)\left(\beta_{2}+g\right)}\theta^{*}E_{A}E_{S}k\right]\\ & \leq \frac{\gamma_{2}{}^{*}}{\beta_{2}+g}\left(\frac{\alpha_{1}{}^{*}Ik}{\beta_{1}+g}-1\right)+\frac{\gamma_{2}{}^{*}\tau^{*}R_{A}k}{(\beta_{1}+g)\left(\beta_{2}+g\right)}\left(1-m\right)\\ & -\frac{\gamma_{2}{}^{*}\gamma_{1}{}^{*}R_{S}k}{(\beta_{1}+g)\left(\beta_{2}+g\right)}+\frac{m\tau^{*}}{\beta_{1}+g}\left(\frac{\alpha_{2}{}^{*}Ik}{\beta_{2}+g}-1\right)\\ & +\frac{\gamma_{2}{}^{*}-m\tau^{*}}{(\beta_{1}+g)\left(\beta_{2}+g\right)}\theta^{*}E_{A}E_{S}k \end{array}$$

This gives $max\left\{\frac{\alpha_{1}{}^{*}k}{\beta_{1}+g},\frac{\alpha_{2}{}^{*}k}{\beta_{2}+g}\right\}\leq 1$ and $\frac{dL}{dt}\leq 0$ if $R_{0}$ ≤ 1. Therefore, LaSalle’s invariance principle implies that the infection-free equilibrium point ${Ε}_{0}$ (1,0,0,0,0) is global asymptotically stable when $R_{0}$ ≤ 1.

**Theorem** **A4.**
*To block the dissemination of event-related rumors input by deviant advocates from the source during the period of social psychological alarm reaction, so that there will be no deviant supporters and amplifiers. Then, the intensity of the intervention $\bar{\alpha_{1}}$ and $\bar{\alpha_{2}}$ should satisfy certain constraints at the same time: $\bar{\alpha_{1}}\geq \alpha_{1}-\frac{\left(\beta_{1}+g\right)}{k}$ and $\bar{\alpha_{2}}\geq \alpha_{2}-\frac{\left(\beta_{2}+g\right)}{k}$.*

**Proof** **of** **Theorem A4.** Based on Theorem A2 and Theorem A3, it is clear that when the infection-free equilibrium point ${Ε}_{0}$ (1,0,0,0,0) is both locally and globally stable, the sufficient condition is $R_{0}$ ≤ 1, that is to say, $max\left\{\frac{\alpha_{1}{}^{*}k}{\beta_{1}+g},\frac{\alpha_{2}{}^{*}k}{\beta_{2}+g}\right\}\leq 1$. Obviously, $\frac{\alpha_{1}{}^{*}k}{\beta_{1}+g}\leq 1$ and $\frac{\alpha_{2}{}^{*}k}{\beta_{2}+g}\leq 1$, that is, $\frac{\left(\alpha_{1}-\bar{\alpha_{1}}\right)k}{\beta_{1}+g}\leq 1$ and $\frac{\left(\alpha_{2}-\bar{\alpha_{2}}\right)k}{\beta_{2}+g}\leq 1$. It can be written as $\bar{\alpha_{1}}\geq \alpha_{1}-\frac{\left(\beta_{1}+g\right)}{k}$ and $\bar{\alpha_{2}}\geq \alpha_{2}-\frac{\left(\beta_{2}+g\right)}{k}$. Hence, Theorem A4 is proved. □

**Theorem** **A5.**
*In the period of social psychological alarm reaction, the intensity of the intervention $\bar{\alpha_{1}}$ and $\bar{\alpha_{2}}$ significantly affect the final size of supporters and amplifiers R(∞), and $R\left(\infty \right)=1-e^{\ln I\left(0\right)-\Delta }$. Here, $\Delta =\left\{max\left\{\frac{\left(\alpha_{1}-\bar{\alpha_{1}}\right)k}{\beta_{1}+g},\frac{\left(\alpha_{2}-\bar{\alpha_{2}}\right)k}{\beta_{2}+g}\right\}\right\}\frac{I\left(0\right)+R\left(\infty \right)-1}{I\left(0\right)}+\frac{\left(\alpha_{1}-\bar{\alpha_{1}}\right)k}{\beta_{1}+g}E_{S}\left(0\right)+\frac{\left(\alpha_{2}-\bar{\alpha_{2}}\right)k}{\beta_{2}+g}E_{A}\left(0\right)$.*

**Proof** **of** **Theorem A5.** The state transition and evolution process of supporters and amplifiers under the rumor intervention is also a process of guiding deviant engagers to gradually become benign. We can understand that when the social network system reaches an equilibrium state, all deviant supporters and amplifiers will eventually die out, that is to say, there are only ignorant, immune 1 and immune 2 left in the system. In that situation, we analyze the final size of ignorant $R\left(\infty \right)=R_{S}\left(\infty \right)+R_{A}\left(\infty \right)$, which can be employed to measure the level of rumor intervention influence. Since our system may be a bit complicated, we calculate I(∞), which means the number of individuals who have not heard of the deviant public opinion in the end. Actually, R(∞)=1−I(∞). According to Arino et al. [48], the final size relation involves the basic reproduction number $R_{0}$ and the n×n matrix V, which describes the transitions between infected (deviant engagement) states and removals from infected (deviant engagement) states. Following Driessche and Watmough [49], we note that only $E_{S}\left(t\right)$ and $E_{A}\left(t\right)$ are involved in the calculation of V. So, we can have

(A7) $$V=\left(\begin{array}{cc} \beta_{1}+g & 0\\ 0 & \beta_{2}+g \end{array}\right),V^{-1}=\left(\begin{array}{cc} \frac{1}{\beta_{1}+g} & 0\\ 0 & \frac{1}{\beta_{2}+g} \end{array}\right)$$

 □

From the concepts presented in [34], the final size of the ignorant is

(A8) $$\ln\frac{I\left(0\right)}{I\left(\infty \right)}=R_{0}\frac{I\left(0\right)-I\left(\infty \right)}{I\left(0\right)}+\left(\alpha_{1}{}^{*}k,\alpha_{2}{}^{*}k\right)\left(\begin{array}{cc} \frac{1}{\beta_{1}+g} & 0\\ 0 & \frac{1}{\beta_{2}+g} \end{array}\right)\left(\begin{array}{c} E_{S}\left(0\right)\\ E_{A}\left(0\right) \end{array}\right)\;=R_{0}\frac{I\left(0\right)-I\left(\infty \right)}{I\left(0\right)}+\frac{\alpha_{1}{}^{*}k}{\beta_{1}+g}E_{S}\left(0\right)+\frac{\alpha_{2}{}^{*}k}{\beta_{2}+g}E_{A}\left(0\right)$$

Putting $R_{0}=max\left\{\frac{\left(\alpha_{1}-\bar{\alpha_{1}}\right)k}{\beta_{1}+g},\;\frac{\left(\alpha_{2}-\bar{\alpha_{2}}\right)k}{\beta_{2}+g}\right\}$ and R(∞)=1−I(∞) into the above formula and using the logarithm algorithm, we can obtain that $R\left(\infty \right)=1-e^{\ln I\left(0\right)-\Delta }$. Thus, Theorem A5 is proved.
